# ChoCo: a Chord Corpus and a Data Transformation Workflow for Musical Harmony Knowledge Graphs

**DOI:** 10.1038/s41597-023-02410-w

**Published:** 2023-09-20

**Authors:** Jacopo de Berardinis, Albert Meroño-Peñuela, Andrea Poltronieri, Valentina Presutti

**Affiliations:** 1 Department of Informatics, King’s College London, 30 Aldwych, London, WC2B 4BG UK; 2 Department of Computer Science and Engineering, University of Bologna, Mura Anteo Zamboni, 7, Bologna, 40126 Italy; 3 LILEC, University of Bologna, Via Cartoleria, 5, Bologna, 40124 Italy

**Keywords:** Computer science, Information technology, Arts

## Abstract

Various disconnected chord datasets are currently available for music analysis and information retrieval, but they are often limited by either their size, non-openness, lack of timed information, and interoperability. Together with the lack of overlapping repertoire coverage, this limits cross-corpus studies on harmony over time and across genres, and hampers research in computational music analysis (chord recognition, pattern mining, computational creativity), which needs access to large datasets. We contribute to address this gap, by releasing the Chord Corpus (ChoCo), a large-scale dataset that *semantically* integrates harmonic data from 18 different sources using heterogeneous representations and formats (Harte, Leadsheet, Roman numerals, ABC, etc.). We rely on JAMS (JSON Annotated Music Specification), a popular data structure for annotations in Music Information Retrieval, to represent and enrich chord-related information (chord, key, mode, etc.) in a uniform way. To achieve semantic integration, we design a novel ontology for modelling music annotations and the entities they involve (artists, scores, etc.), and we build a 30M-triple knowledge graph, including 4 K+ links to other datasets (MIDI-LD, LED).

## Background & Summary

Western tonal music encompasses several dimensions (melody, harmony, rhythm, etc.) and temporal scales (beat, measure, phrase, etc.), which all contribute to characterise a complex signal studied in different fields and from various lenses. One prominent dimension is represented by harmony, also known as the “vertical dimension” of music, which is concerned with “*combining notes in music to produce a pleasing effect greater than the sum of its parts*”^1^. Harmony is a widely studied component in music theory^2,3^, and music analysis^4^; where functional harmony provides a set of rules for moving to and from the *tonic* – the most stable note in a piece, allowing to relate chords to each other, and to the main harmony.

Chords are the basic constituents of harmony, which jointly define the harmonic structure of a piece. Individually, a chord is defined as a simultaneous occurrence of several music sounds, producing harmony^5^. Depending on the notational system and the annotation conventions, a chord can be associated with a name, or label. For example, the chord G7 (typically read as “*G dominant seventh*”) in the key of C major, contains the notes G-B-D-F and may create tension partly due to the tritone relation between B (leading tone) and F (the seventh of the chord). These intervals to the root characterise the intrinsic harmonic properties of chords, as well as the relationships with other chords in the same harmonic progression^6^.

Perceptually, some chords sound more stable, final and resolved, while others sound unstable and tense – a phenomenon that is salient both to young children and to adults, even from diverse cultures. However, the definition of harmony differs vastly across time, genre, and individuals^7^, reflecting a great heterogeneity in terms of harmony perception^8,9^; and in this work, we focus on Western tonal music tradition. In this regard, harmony exerts an affective role: major harmonies tend to represent positive emotions (happiness, joy, triumph, etc.); minor triads express “negative” emotions (sadness, anger, etc.); diminished triads (chains of minor thirds) indicate suspense and other disorienting sentiments, while augmented triads (all major third intervals) tend to create senses of spookiness, extreme dark emotions, and mystery^1^.

Computationally, the automatic analysis of chord progressions has addressed several tasks in information retrieval – from the detection of cadences, patterns, structures in music, to the introduction of harmonic similarity measures for cover song detection, symbolic search, and content-based music linking. Progress in machine learning research has also sparked interest in computational creativity applications, such as arrangement generation, continuation, infilling, and automatic music composition with harmonic conditioning^10^ (e.g. generating melodies from a given harmonic template) to name a few.

To account for the evolution of harmony and explain its subjective and genre-specific differences, while enabling the aforementioned applications, the availability of large, diverse, and reliable chord data is fundamental. However, several different chord notations exist (Harte, Roman, ABC, Leadsheet, etc.), each with different levels of expressiveness, in a large number of disconnected chord datasets that are hard to combine^11^. This poses a challenge for combining existing chord datasets into larger ones. Existing approaches address this issue by focusing on scale, and publishing large numbers of chord annotations. For example, UltimateGuitar (https://www.ultimate-guitar.com/) offers a collection of 1.1 M + songs annotated by a community of 12 M + musicians. Chordify (https://chordify.net) addresses the challenge of scalable chord annotation by applying methods for automated chord estimation. However, none of these approaches solves the problem of integrating chord datasets complying with the following desiderata: (**a**) high quality of the data; (**b**) precise timing information; (**c**) release through open licences; (**d**) use of different chord notations; (**e**) diversity of music genres; and (**f**) large scale. The problem is exacerbated by the little reuse of standard formats for music annotation. In the context of this article, *music annotation* is defined, in a broad sense, as the outcome of a music analysis carried out by a domain expert on the musical surface (a score, a recording) to identify and locate elements of interest (e.g. chords, segments, patterns, etc.), following an established methodology. For example, if the goal of a harmonic analysis is to identify chords from a composition, a music annotation may correspond to a list of chords together with a reference to their onset and offset (i.e. when they occur in the piece).

### The problem of music data scarcity and interoperability

In the last decade, numerous systems and formats have been proposed for representing and storing musical annotations^12^. Some have been more successful than others, but no system has prevailed as a reference standard. Some systems are focused on symbolic music and are domain-specific (e.g. DCMLab, RomanText for harmonic analyses), embed annotations in the score (MusicXML, ABC, etc.), or propose variations of tabular formats to account for audio and symbolic music (LAB and xLAB). In the audio domain, JAMS (JSON Annotated Music Specification)^13^ has emerged as a system to uniformly represent music annotations of different types and granularity, that is efficiently built on top of the JSON serialisation standard. JAMS is also supported by software libraries for dataset manipulation^14^ and for the evaluation of MIR methods^15^.

However, combined efforts of MIR and Semantic Web (SW) researchers to address (chord) annotation data interoperability have been scarce. While MIR has contributed a great deal of music datasets, predominantly containing music annotations to train and evaluate computational methods for music analysis, SW technologies and principles can easily address the data integration problem at scale^11^. Nevertheless, the scarcity of semantic models for music annotations has hampered this vision, and more research efforts are hence necessary to devise domain-specific ontologies that can efficaciously address the interoperability issue through reuse and alignment. In addition, this kind of musical knowledge is also underrepresented in Knowledge Graphs^16^, which are usually built from other knowledge archetypes such as logic statements or textual corpora. The lack of musical knowledge in the Semantic Web also limits our understanding of knowledge expressed in modalities other than text (e.g. images, music) and its challenges: semantic relations that have not been formalised yet, integration of multimodal datasets, etc.

Specifically for harmonic data, various chord collections have been published (see Table 1) making harmony annotations available, albeit through highly heterogeneous and non-interoperable notations (Harte, Leadsheet, Roman, ABC) and formats (JAMS, JSON, MusicXML, LAB, etc.). Other databases, such as UltimateGuitar and Chordify^17^, focus on automation and scalability. These are achieved by annotating millions of songs via crowdsourcing or chord recognition algorithms, but have an inherent cost in annotation quality. Therefore, none of these approaches solves the problem of semantically integrating chord annotation datasets while meeting all the aforementioned desiderata (*a*-*f*).

**Table 1:** Overview of the 18 chord datasets currently included in ChoCo. Letters “A” and “S” are used to denote *audio* and *symbolic* (or *score*) music subsets, respectively – from which harmonic annotations are collected.

Collection	Type	Notation	Original format	Annotations	Genres	Ref
Isophonics	A	Harte	LAB	300	pop, rock	^31^
JAAH	A	Harte	JSON	113	jazz	^38^
Schubert-Winterreise	A, S	Harte	csv	25 (S), 25*9 (A)	classical	^39^
Billboard	A	Harte	LAB, txt	890 (740)	pop	^32^
Chordify	A	Harte	JAMS	50*4	pop	^7^
Robbie Williams	A	Harte	LAB, txt	61	pop	^33^
The Real Book	S	Harte	LAB	2486	jazz	^36^
Uspop 2002	A	Harte	LAB	195	pop	^34^
RWC-Pop	A	Harte	LAB	100	pop	^35^
Weimar Jazz Database	A	Leadsheet	SQL	456	jazz	^41^
Wikifonia	S	Leadsheet	mxl	6500+	various	—
iReal Pro	S	Leadsheet	iReal	2000+	various	—
Band-in-a-Box	S	Leadsheet	mgu, sku	5000+	various	^42^
When in Rome	S	Roman	RomanText	450	classical	^44^
Rock Corpus	S	Roman	har	200	rock	^47^
Mozart Piano Sonata	S	Roman	DCMLab	54 (18)	classical	^4^
Jazz Corpus	S	Hybrid	txt	76	jazz	^49^
Nottingham	S	ABC	ABC	1000+	folk	^48^

The challenge of supporting interoperability of music content-related data has been the subject of relevant efforts in the last decade, especially supporting their evolution, reuse, and sustainability^18–20^ according to FAIR data principles^21^ and through Semantic Web technologies. The Music Ontology^22^ addresses contextual metadata about music pieces, such as when they were recorded or arranged and by whom, providing a basis for interlinking music datasets. The OMRAS2 Chord Ontology^23^ defines a vocabulary to describe chords and chord sequences in RDF, albeit is no longer maintained and does not support reasoning. The HaMSE Ontology^24^ aligns different music representation systems and describes a set of musicological features to enable different degrees of interoperability. MusicOWL^25^ describes the structure of music scores to support MIR tasks using musical features rather than text. It addresses concepts such as clef, dynamic, key, note, etc., and reuses the Tonality Ontology (http://motools.sourceforge.net/doc/tonality.html) on top of the Music and Chord ontologies. The Music Theory Ontology (MTO)^26^ adds music theoretical notions that were overlooked in existing ontologies or only partly defined, such as musical notation, duration, progression and degree, aligning with e.g. the Chord Ontology and supporting deductive inferences such as scale degree and chord degree. The Diatonic-Chromatic System Ontology^27^ uses reasoning to infer if a score can be classified within the analytical framework of Michael Praetorious (1571–1621). A lightweight RDF vocabulary for representing events contained in MIDI files has been proposed with the MIDI Ontology^28^.

Some of these ontologies are the backbone of large music notation knowledge graphs. For example, the MIDI Ontology^28^ has been used to generate the MIDI Linked Data Cloud (https://midi-ld.github.io/), a large knowledge graph interconnecting 300 K+ MIDI files through 10B+ triples of music-related linked data addressing music content rather than metadata. This misses, however, explicit chord information that could be useful for the symbolic analysis of harmony. MusicOWL^25^ has been used for producing the Linked Music Score Dataset (https://linkeddata.uni-muenster.de/datasets/opendata/ulb/musicscores/) knowledge graph, representing elements of 43 historical scores from the Münster University Library. Yet, none of these previous efforts successfully addresses the challenges *a*-*f*); especially providing representations that meet the standards and the needs of different communities (e.g. JAMS for MIR, Musicology, and RDF for Semantic Web, Digital Humanities, etc).

### Our contribution

We present the **Chord Corpus** (**ChoCo**)^29^, a large dataset for musical harmony knowledge graphs. We describe the data workflow to curate, transform, and integrate more than 20,000 human-made, high-quality harmonic annotations from 18 highly heterogeneous chord datasets (desiderata *a*, *b*, *f*), following the JAMS data structure as annotation model. The resulting annotations are rich in provenance data (e.g. metadata of the annotated work, authors of annotations, identifiers, etc.) and refer to both symbolic music notation and audio recordings, while encompassing different notation systems (desideratum *d*). After semantically enriching, extending, and standardising these annotations under the JAMS definition, we use our ontologies to release the ChoCo Knowledge Graph – providing fine-grained semantic descriptions of chords, opportunities for chord interoperability, and 4 K+ links to external datasets. All data and code are released using open data licences (desideratum *c*). We also show evidence of interest and use of ChoCo, and postulate its value for the Semantic Web and MIR communities at enabling the study of harmony through large scale data. Specifically, the main contributions are summarised as follows.

- A large dataset and knowledge graph standardising, enriching, and integrating 18 existing chord collections in the literature. ChoCo is released both as a JAMS dataset and an RDF knowledge graph, to accommodate the requirements and needs of different communities (Music Information Retrieval, Musicology, Semantic Web, etc.).
- A generalised data curation framework to semantically integrate MIR harmonic datasets and represent chords from a large variety of formats (JSON, CSV, LAB, TXT, SQL, MusicXML, iReal, mgu, sku, ABC, etc.) as JAMS annotations.
- An ontological and extensible model to represent JAMS annotations, and chord annotations in particular, as RDF knowledge graphs, together with a set of SPARQL queries to extract chord-related information from JAMS files directly or transform them into RDF, using state of the art data engineering methods.

ChoCo achieves interoperability of harmonic datasets at three levels: metadata, annotation format, and chord notation. The interoperability at metadata and annotation format levels is implemented by integrating metadata from different sources, at the parsing level, and by leveraging the JAMS annotation standard to store harmonic annotations, consistently. Chord notation interoperability is achieved by converting chords to three reference notational systems (desideratum *d*) – bridging them via the Harte notation^30^. The outcome of this approach enables the use of these integrated collections as if they belonged to the same dataset and underpins the automatic generation of Music Knowledge Graphs. In addition to the conversions, ChoCo provides the original annotations in each JAMS file, along with rich provenance descriptions that keep track of the original sources.

## Methods

**Fig. 1:**
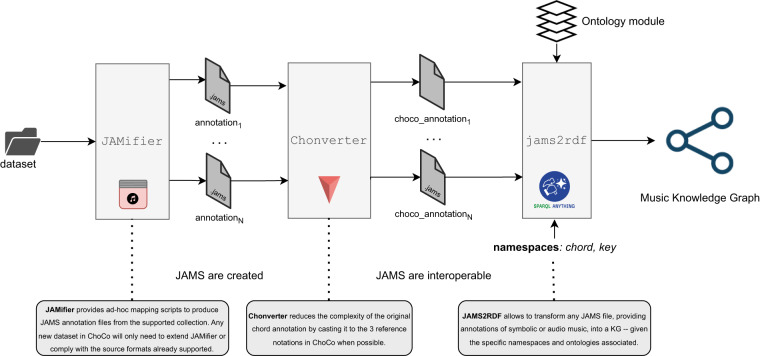
Overview of our data transformation workflow, generalised for arbitrary music annotations, and used here for chord and key annotations prior to the generation of the ChoCo Knowledge Graph. The JAMifier ingests chord collections (where metadata and music annotations follow collection-specific conventions and formats) to generate a JAMS dataset. This achieves two integration levels, as all *metadata* are consistently re-organised, and the *music annotations* (i.e. chord progressions, in this case) are all encoded and stored in separate JAMS files – one per track/score. The Chonverter achieves notational interoperability among collections by converting the original annotations to the same notational families. Finally, jams2rdf leverages notation-specific ontologies to generate RDF triples and create a Music Knowledge Graph.

The general workflow to produce ChoCo is illustrated in Fig. 1. We describe the resources contained in ChoCo, and the data transformation workflow to: produce JAMS datasets (Jamifier), integrate the different chord notations (Chonverter), and create a music knowledge graph.

### Chordal data in ChoCo

**Fig. 2:**
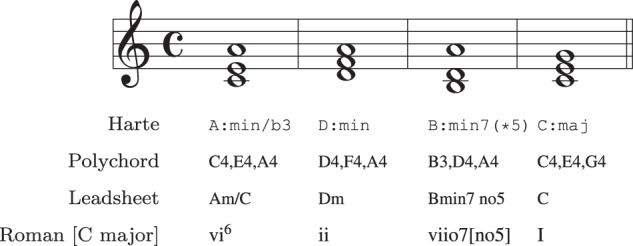
Example of a harmonic progression annotated using different notation systems, namely (i) Harte, (ii) Polychord (or decomposed chords), (iii) Leadsheet, and (iv) Roman Numerals.

Table 1 summarises the source chord datasets (alias *subsets*, *collections*) that are integrated in our framework. ChoCo v1.0 integrates 18 high-quality chord datasets providing timed annotations of chord progressions in different formats (e.g. LAB, CSV, txt, mxl), notations (e.g. Harte, Leadsheet, Roman, ABC), and types (audio, symbolic). The rich and diversified nature of this resource, encompassing several genres/styles and periods, makes it the largest chord collection of its kind – with more than 20 K annotated progressions. ChoCo’s collections can be categorised according to their generalised chord notation system: Harte, Polychord, Leadsheet, and Roman. An example of notation systems for the same chord progression is given in Fig. 2.

#### Harte collections

This group gathers  all collections with chords expressed in Harte notation^30^. The majority of these datasets are focused on pop/rock music, released in LAB format, and collected from audio music. Among them, *Isophonics*^31^ provides chord, key, and structural annotations of a selection of albums by The Beatles, Queen, Michael Jackson, and Carole King; *Billboard*^32^ contributes similar annotations for a collection of songs sampled from the Billboard “Hot 100” chart in the United States between 1958 and 1991; *Chordify Annotator Subjectivity Dataset (CASD)*^7^ augments a subset of Billboard with 4 expert annotators per song – to demonstrate the highly subjective nature of the chord identification/labelling task; *Robbie Williams*^33^ contains key and chord annotations for 5 albums from this artist; *Uspop2002*^34^ is a large scale dataset for music similarity, providing audio features, style tags, artist similarity ratings, as well as harmonic annotations for a smaller subset; *RWC-Pop* is a subset of the the Real World Computing (RWC) database^35^, a cornerstone collection in MIR containing a great deal of instrumental and performance annotations, in addition to chordal information that was contributed by LabROSA. Among the other (non-pop) collections, we find the *Real Book*^36^, providing chord annotations of several jazz standards from the homonymous book^37^; the *Audio-Aligned Jazz Harmony* (JAAH) dataset^38^ contributing time-aligned harmony transcriptions from “The Smithsonian Collection of Classic Jazz” and “Jazz: The Smithsonian Anthology”; and finally, the *Schubert Winterreise*^39^ multi-modal dataset, containing harmony and segment information of Franz Schubert’s song cycle “*Winterreise*” which were separately annotated from the score and from the audio (9 performances per score).

#### Leadsheet collections

Four ChoCo collections use different flavours of the Leadsheet notation^40^ for a variety of genres. These include the *Weimar Jazz Database*^41^, providing rich cataloguing information, scores, YouTube links, and harmonic/melodic annotations of a selection of jazz solo transcriptions; *Wikifonia*, a copyright-free online publisher of sheet music in MusicXML format which was discontinued in 2013; the *Band-in-a-Box (BiaB) Internet corpus*^42^, containing human-generated chord annotations for BiaB – a commercial software (https://www.pgmusic.com) that is used to generate accompaniment for musical practice; the *iReal Pro* collection, a newly contributed chord dataset of various genres (jazz, blues, brazilian, latin, country, pop) that was created from the public playlists of iReal Pro (https://www.irealpro.com) – a commercial app with similar functionalities to BiaB.

#### Roman collections

These include  chord datasets providing harmonic annotations in Roman notation^43^, and with more emphasis on classical music. A central dataset here is *When in Rome*^44^, which already contains harmonic analyses from the *TAVERN* collection^45^ (theme and variations for piano by Mozart and Beethoven), and the *BPS-FH* dataset^46^ (Beethoven piano sonata); but also harmonic annotations from Monteverdi madrigals, Bach chorales and preludes, Haydn Op. 20 String Quartets, and a subset of nineteenth-century songs from the OpenScore Lieder corpus (Winterreise and Schwanengesang cycles from Schubert, Dichterliebe from Schumann, and several pieces by female composers). Notably, *When in Rome* is an actively maintained corpus where new harmonic annotations (in RomanText format) are also contributed and internally validated by experts. As a growing corpus of functional harmonic analyses, we plan to support the integration of future releases within ChoCo. Other Roman collections include the *Rock Corpus*^47^, providing harmonic analyses, melodic transcriptions and lyrics information produced from a sample of Rolling Stone magazine’s list of the “500 Greatest Songs of All Time” in 2004 (pages 65–165); and *Mozart Piano Sonata*^4^, featuring harmonic, phrase, and cadence analyses of all piano sonatas by Mozart.

#### Other collections

ChoCo also includes *Nottingham*^48^, a dataset of British and American folk tunes, (hornpipe, jigs, etc.) released in ABC format; and the *Jazz Corpus*^49^, providing harmonic analyses of jazz standards using both Harte-like and functional notations, the latter of which is akin, in purpose, to Roman numerals.

#### Chord datasets not included in ChoCo

Although other collections providing harmonic information exist in the literature, some of them were currently discarded for the reasons explained below. The *Leadsheet* dataset^50^ separately annotates chord progressions for each segment (e.g. intro, chorus) but does not provide information on how structures are laid out in the piece. *GuitarSet*^51^ only provides 3 unique (and short) chord progressions. *UMA-Piano*^52^ only contains audio recordings of chords, played independently. Finally, *POP909*^53^ and the *Kostka-Payne* corpus^54^ provide computationally-extracted chords and keys, whereas the first release of ChoCo focuses on high-quality annotations for time being.

### From chordal data to JAMS datasets

The first challenge of bringing together existing chord datasets into a coherent, uniform corpus is the variety of formats in which chord annotations, and other related information, are encoded. In order to address this issue, we use JAMS data structure^13^ as a simple, content-agnostic wrapper for expressing music annotations in general, and chord annotations in particular. JAMS relies on the popular Web data exchange JSON format, and enforces the following structure based on three basic properties (see https://jams.readthedocs.io/ for additional details):

- file_metadata, describing the music piece these annotations refer to. More precisely, it contains these properties: identifiers, optionally providing explicit links to external resources, mostly relating to cataloguing information from online music databases, e.g., MusicBrainz (https://musicbrainz.org); artist, referring to a performer or a band; title of the musical work; release, intended as a more general definition of album; and duration, defining a temporal span within which annotations can fall.
- annotations, a container of annotation objects, each describing a specific *namespace* (the term *namespace* in JAMS has a different sense than a Web namespace) that identifies the type of the annotation’s subject (e.g., chords, structural segments, emotions, patterns, keys, etc.). These annotations also include metadata to document the annotation process (e.g. whether the annotation is manually produced or inferred by an algorithmic method, the name of the annotator or software, information about the annotation tools, rules and validation).
- sandbox, described as an unrestricted place to store any additional data.

Listings 1 and 2 show excerpts of an example JAMS file from the Isophonics collection^31^ annotating chords for Queen’s *Bohemian Rhapsody*, taken from the Isophonics collection.

Although JAMS has an implicit focus for audio-based annotations, its definition and structure are flexible enough to be easily extendable to the symbolic domain. This is also confirmed by the modular design of the codebase, where additional namespaces can be registered by a user, by simply providing regular expressions to validate the annotation content (e.g. a new chord notation). In other words, any arbitrary music annotation can be described within JAMS as long as the atomic observations (e.g. the individual occurrences of chords making up the progression) are described in terms of: time, a temporal anchor specifying the onset of the observation; duration, value (e.g. *Bb:maj7*), and confidence, a scalar in [0, 1] expressing a level of certainty by the annotator (or algorithm). Therefore, the only elements distinguishing audio from symbolic annotations, are the temporal specifications (time and duration), which are described in absolute (seconds) or metrical (measure and beat/offset) terms, respectively. For symbolic annotations, we number measures and beats from 1 for convenience, without attempting to emulate exact musical (editorial) practice for cases like anacrustic openings.

**Listing 1**. Excerpt of the three first chords in a JAMS file annotating Queen’s Bohemian Rhapsody.

**Listing 2**. Annotation and file metadata in a JAMS file annotating Queen’s Bohemian Rhapsody.

#### JAMification of datasets

Considering the diversity of annotation formats and conventions for data organisation (the way content is scattered across folders, files, database tables, etc.), each chord dataset in ChoCo (c.f. Table 1) undergoes a standardisation process lending to the creation of a JAMS dataset. This is needed to aggregate all relevant annotations of a piece (chord, key, etc.) in a single JAMS file, and to extract content metadata from the relevant sources.

The content metadata of a (music) dataset is indeed crucial to identify, describe and retrieve the actual musical content being annotated. This typically includes the title of each piece, artists (composers and/or performers), and cataloguing information (album/release or collected work), ideally with the provision of identifiers (e.g. MusicBrainz IDs). Nevertheless, only the *Mozart Piano Sonata* collection^4^ provides complete content metadata in a csv file, as usually expected from a music dataset. When content metadata is missing, this may be found online (HTML pages, supplementary material), from articles/reports documenting the collection, by resolving any cross-reference among files and dataset-specific identifiers, extracted from the actual score (or better, the dataset-specific representation of the score). Alternatively metadata can be derived from the organisation of files in folders. For example, Michael Jackson/Essential Michael Jackson [Disc 01]/1-16_Beat_it.lab indicates author, album, disc, track number and title, respectively. This organisation varies as the datasets vary – a consequence of the lack of a standard “datasheet for datasets” in the music domain^55^.

The same issue applies to the extraction, pre-processing, and standardisation of harmonic annotations from these collections, some of which were never released as chord datasets (*Weimar Jazz Database*, *Wikifonia*, *iReal Pro*, *Nottingham*). Harmonic annotations can be encoded in different formats (LAB, XLAB, RomanText, CSV, DCMLab, JSON, SQL, TXT), or extracted from symbolic music (MusicXML, ABC) and backing tracks in proprietary encodings (iReal, MGU). As each collection shows a specific combination of the mentioned issues (different organisation of content and metadata, different annotation formats and conventions), this step required considerable effort. The result of this standardisation process may improve the usability of these resources for music researchers, and simplifies the KG construction process. In addition, for the symbolic subsets, we also include time signatures (initial time signature and subsequent metrical changes) as annotations in each JAMS file (using a dedicated timesig namespace); which makes it easier to interpret the temporality of each chord (onset and duration) at hand.

Following the standardisation process, each of these 18 JAMS datasets represents a novel contribution per se, due to the heterogeneity of annotation formats and practices, and the limited availability of content metadata in their original version. This also includes *CASD*, a collection that provides chords in JAMS format, but lacks local key annotations, which were retrieved from *Billboard* (we remind that CASD is already a subset of Billboard).

#### Conversion of chord notations

As shown in Table 1, the third element of divergence besides annotation formats and provision of content metadata, is the notation system used to represent chords. To address this issue we perform the following actions: (i) decomposition of domain-specific notations to chord constituting elements; (ii) conversion of the decomposed chord to the Harte framework; (iii) conversion of chord progressions by iteratively applying steps (i) and (ii) to all the chords in a sequence/progression. This yields a new JAMS file with the converted chord annotations.

For all the above steps, specific software was developed for processing the different annotation types contained in the original datasets. There are three main types of chords that are processed: Roman Numerals chords (e.g. C minor:viio7/V), Polychords (e.g. E4,G#4,B4), Leadsheet chords (e.g. Gm7/F). With *Leadsheet chords* we refer to a broader category, although each dataset using this format proposes a different flavour of this notation. For example, a G minor chord in *Wikifonia* is annotated as G min, whereas the same chord is annotated as G- in the *Jazz-corpus*.

**Fig. 3:**
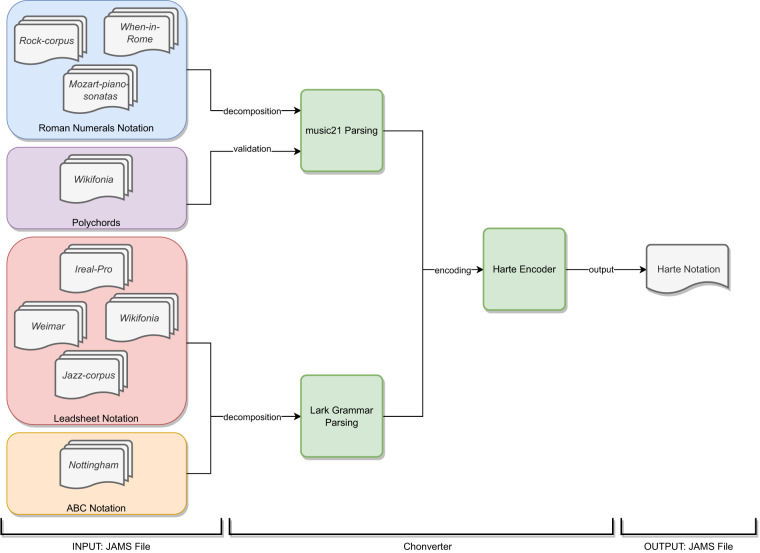
Overview of the Chonverter workflow, describing how different chord notations are converted to the Harte notation.

As outlined in Table 1, each dataset uses a flavour of the same notation to represent chords, with the exception of *Wikifonia*, where some annotations use both Leadsheet and Polychords even for the same progression; and the Jazz Corpus, providing chords encoded in both Roman Numerals and Leadsheet. Figure 3 provides a taxonomy of the different notational flavours, together with a schematic overview of the conversion workflow.

In step (i), a chord is first decomposed into its components (e.g. C major → C, E, G). For this purpose, the Chonverter uses a family of tools depending on the source notation. Roman numerals are decomposed using the *roman module* of music21^56^, a Python library for computational musicology. As Polychords already provide note constituents by definition, this step is limited to preprocessing the symbols associated to the different pitches in a chord. Polychords are usually mixed with chords annotated in other notations (e.g. Leadsheet), so it is necessary to differentiate the type of chords when parsing. Finally, for each Leadsheet flavour, a context free grammar was created to parse the original annotation of the chord. A different grammar was created for each dataset containing annotations in leadsheet format, namely *Weimar Jazz Database*, *Wikifonia*, and *iReal Pro*, using the Lark library (https://github.com/lark-parser/lark). Notably, the ABC notation used in *Nottingham* is similar to the Leadsheet notation and was therefore processed in the same way. This process is more intuitive for all collections natively using the Harte notation, as the latter already accounts for the description of chord pitches^30^.

After all chords are decomposed as lists of pitches, it is then possible to associate a shorthand (a string) to each list according to the Harte notation (Step (ii)). The Chonverter achieves this via music21 and defines rules for composing Harte chords.

New JAMS files are produced after the last step, each providing a new annotation (with chord_harte as namespace). Whenever an original annotation uses *Leadsheet* or *Polychord* notations, the new annotation replaces the original, since the conversion provides a generalisation of the different flavours via a syntactic transformation. Instead, if the original annotation contains *Roman Numerals* chords, the new (converted) annotation is added to the existing one, since the Roman Numerals contain information that would otherwise be lost, i.e. the harmonic functions that the chords hold within the piece.

The Chonverter module performs a syntactic conversion of chord labels. However, converting Roman Numeral also requires taking into account the key of the song. Moreover, a distinction has to be made between key-relative and absolute chords. Some music is always played in the same key, while other pieces are frequently transposed. For example, symphonies are often performed in a fixed key, while lieder are typically performed in multiple keys depending on the singer’s vocal range. Datasets like *When in Rome* contain transcriptions of these key-flexible works. Even in these cases, chords in ChoCo are always converted by taking into account the tonality provided by the original dataset for that piece. However, whenever this happens, the generated conversion, although correct, may only be one of several possible conversions.

### The JAMS Ontology and the ChoCo Knowledge Graph

To represent JAMS annotations as linked data (LD) we designed an ontology that formally represents the JAMS data model. The JAMS Ontology is part of the Polifonia Ontology Network (PON, https://github.com/polifonia-project/ontology-network), from which we reused 4 ontology modules (*Core*, *Music Meta*, *Music Representation* and *Music Projection*). Table 2 provides links to ChoCo’s resources, including the JAMS Ontology and the Knowledge Graph (KG).

**Table 2:** Links to key ChoCo resources: ontology, datasets, and knowledge graph.

de Berardinis, J. *et al*. (2023). *ChoCo: a Chord Corpus and a Data Transformation Workflow for Musical Harmony Knowledge Graphs*. 10.5281/zenodo.7706751	
Resource	Link
ChoCo dataset	http://w3id.org/polifonia/resource/choco/
Portal page	https://smashub.github.io
JAMS Vocabulary namespace	http://w3id.org/polifonia/ontology/choco/ prefix (jams)
JAMS Resource namespace	http://w3id.org/polifonia/resource/choco/ prefix (pon-res)
Roman Chord Vocabulary namespace	http://w3id.org/polifonia/ontology/roman-chord/ prefix (roman)
GitHub organisation & code	https://github.com/smashub/
Dataset generation code	https://github.com/smashub/choco
Documentation and tutorials	https://smashub.github.io/docs/category/choco-the-chord-corpus
Example data story	https://projects.dharc.unibo.it/melody/choco/chord_corpus_statistics
VoID description	https://github.com/smashub/choco/blob/main/void.ttl
SPARQL endpoint	https://polifonia.disi.unibo.it/choco/query
Zenodo	10.5281/zenodo.7706751

The JAMS Ontology formally defines the semantics of music annotations that are encoded using JAMS. To improve compliance with the ontology and facilitate the generation of linked data, we have established conventions for including relevant information in the creation phase of the JAMS files. In essence, the JAMS Ontology ameliorates the limitations of the current JAMS model, mainly on two fronts: (i) at the level of metadata, enabling the alignment and linking of tracks belonging to different datasets, and also, with external resources available on the Web; (ii) at the annotation level, allowing to describe data (e.g. a chord) by semantically annotating its components (e.g. root, quality, inversions, etc.) rather than using a label.

Concerning the first level, the JAMS Ontology provides support to trace the original source of an annotation, i.e. whether it refers to a signal representation (audio) or to a symbolic representation (score). This also allows to correctly interpret the content of the annotation. In fact, temporal information is expressed in seconds for audio tracks, whereas it is expressed in beats and measures for symbolic music. This distinction also allows to disambiguate the roles of composers and performers – rather than simply referring to them as “artists”. Furthermore, the alignment with other ontologies makes it possible to describe the musical content of JAMS annotations thereby enabling different types of music analyses.

To achieve this, additional data is dumped by the JAMifier in the *Sandbox* of each JAMS file, and new annotation types were created by contributing new *namespaces*. The JAMS Ontology provides a common conceptual, formal model to interpret JAMS annotations and is available online at the following URI:

https://w3id.org/polifonia/ontology/jams/

Our ontological requirements can be summarised as follows:

- the resulting KG must represent JAMS files and JAMS annotations as such, including their provenance and process-related information: e.g. source dataset, annotator, confidence of each observation, etc;
- the KG must distinguish “performers” (properties of a track/score), from “composers/authors” of a piece (properties of its corresponding musical composition) whenever the data allows for their disambiguation;
- temporal information must be expressed according to the type of the annotation’s subject, i.e. audio or score;
- chords must be represented according to the data model of these notation families: *Harte* and *Roman Numerals*.

To model this ontology, we reused the *Music Annotation Pattern*^57^, an Ontology Design Pattern (ODP)^58^ for modelling different types of music annotations and their related time references. We remark that the terminology used in the JAMS documentation (https://jams.readthedocs.io/en/stable/) is adopted to define the JAMS Ontology vocabulary. In particular, the following terms are (re-)used:

- **Annotation:** an annotation is defined as a group of Observations (see below) that share certain elements, such as the method used and the type of annotation’s subject (e.g. chords, notes, patterns);
- **Observation:** an observation is defined as the content of an annotation, and includes all the elements that characterise the observation. For example, in the case of an annotation containing chords, each observation corresponds to a chord, and specifies, in addition to the chord value, the temporal information and its confidence.

To develop the JAMS Ontology we apply the eXtreme Design methodology^59^ and, according to it, we define a set of competency questions (CQs) that the ontology shall address. These are listed in Table 3. The above CQs were converted into SPARQL queries that served to iteratively test the ontology during its implementation. All SPARQL queries are available in the JAMS Ontology repository (https://github.com/polifonia-project/jams-ontology).

**Table 3:** Competency questions (CQs) addressed by the JAMS Ontology.

ID	Competency question
CQ1	What is the content of the observations contained in a JAMS Annotation?
CQ2	Who is the composer of a musical object?
CQ3	Who is the performer of a musical object?
CQ4	Who/what is the annotator of an annotation/observation, and what is its type?
CQ5	What is the time frame addressed by an annotation, within a musical object?
CQ6	What is its start time (i.e. the starting time of the time frame)?
CQ7	Which are the observations included in an annotation?
CQ8	Given an observation, what is the starting point of the time frame it addresses, within its target musical object?
CQ9	Given an observation, what is its addressed time frame, within its target musical object?
CQ10	What is the key of a composition/performance?
CQ11	What is the value of an observation?
CQ12	What is the confidence of an observation?
CQ13	What are the chords of a composition/performance?

**Fig. 4:**
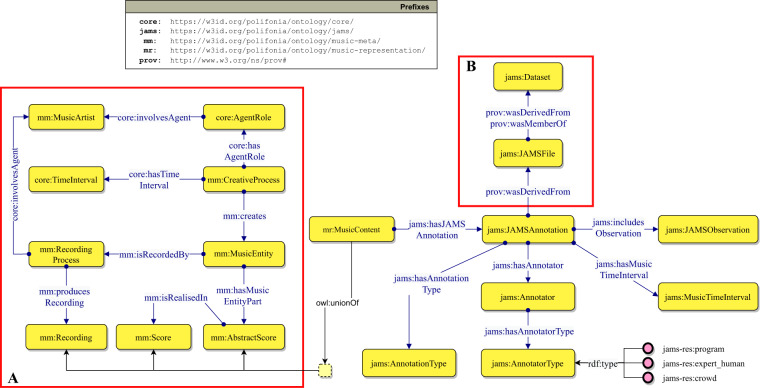
Fragment of the JAMS ontology describing JAMS files and their provenance, musical objects and JAMS annotations. We use the Graffoo notation (https://essepuntato.it/graffoo/): yellow boxes are classes, blue/green arrows are object/datatype properties, purple circles are individuals, green polygons are datatypes.

Figure 4 shows a fragment of the JAMS Ontology modelling a JAMS Annotation. On the left (*box A*), we define the classes and properties for representing the song’s metadata, by reusing the *Music Meta* module from PON. The central class of this ontology module is mm:MusicEntity, an Information Object^60^ defined as the “sum of all the elements” that make up a piece of music. A Music Entity is created by a mm:CreativeProcess, which may represent, for example, the composition process; and can be recorded by a mm:RecordingProcess, which in turn produces a mm:Recording. Both the Recording Process and the Creative Process are related to the agent responsible for the process and its role, represented by the classes mm:MusicArtist and core:AgentRole respectively. Finally, a Music Entity may be associated to an mm:AbstractScore – an abstraction of the score encompassing all the musical characteristics of a composition (e.g. tempo, key, structure) which is then formalised/materialised in one (or more) mm:Score(s). The union of Recording, Score and Abstract Score define a mr:MusicContent, which can be annotated by a jams:JAMSAnnotation.

Here, the Provenance Ontology^61^ is reused to model the provenance of JAMS annotations (Fig. 4, *box B*). Each JAMS annotation derives from a JAMS file (jams:JAMSFile) which is either taken or derived (for example, translated from a file in a different format to the JAMS format) from a dataset jams:Dataset.

**Fig. 5:**
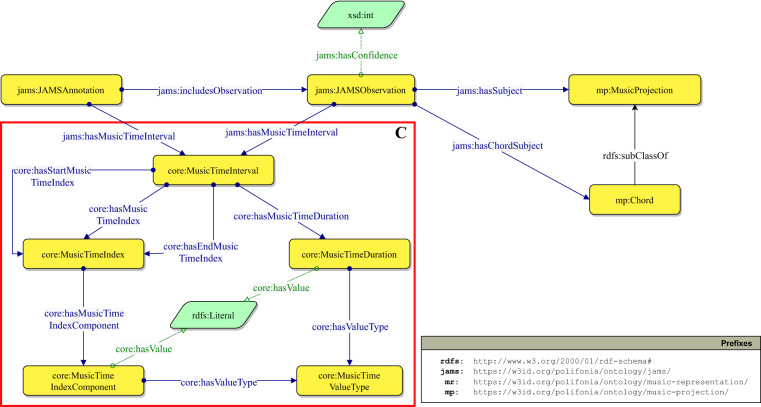
Fragment of the JAMS Ontology describing JAMS annotations and JAMS observations. The red block C highlights how the time information has been modelled for handling different types of formats and standards. We use the Graffoo notation.

A core class of the JAMS Ontology is jams:JAMSAnnotation. It captures the annotation, from a file encoded with the JAMS format, on a musical object (its target): either a recording or a score. A JAMS annotation entity and its musical object are put in relation by means of the property jams:hasJAMSAnnotation. An annotation is performed by an annotator jams:Annotator, has a time validity jams:hasMusicTimeInterval, and contains information of a certain type jams:AnnotationType (e.g. chords, keys, etc.). The validity indicates to which time frame, within a musical object, the annotation refers. For example, if an annotation reports the observation of a certain *key*, that *key* refers to a segment of the target musical object. Annotators may be of different types (e.g. expert annotator, software program), and are defined by the class jams:AnnotatorType. Finally, a jams:JAMSAnnotation is composed of a set of observations jams:JAMSObservation. Figure 5 depicts the JAMS Ontology fragment that models JAMS observations.

A key aspect of observations and annotations is the identification of the musical object fragment they refer to. We model musical object fragments as musical time intervals core:MusicTimeInterval. Musical time intervals can be expressed in different ways, depending on the type of musical object. For example, if the subject of an observation (and in turn of an annotation) is a recording, then we most probably identify its fragments in terms of seconds. If we deal with scores, we may want to use a combination of measures and beats. To make the ontology as flexible as possible for expressing musical time intervals, we model them as being defined by musical time indexes (core:MusicTimeIndex). Each musical time interval has a start time index and an end time index (plus potentially infinite internal time indexes). A musical time index is defined by one or more components (core:MusicTimeIndexComponent), each substantiated by a value (core:hasValue) and a value type (core:MusicTimeValueType). A musical time interval also has a duration (core:MusicTimeDuration) which is expressed by means of a value and a value type (usually seconds for recordings and beats for scores).

**Fig. 6:**
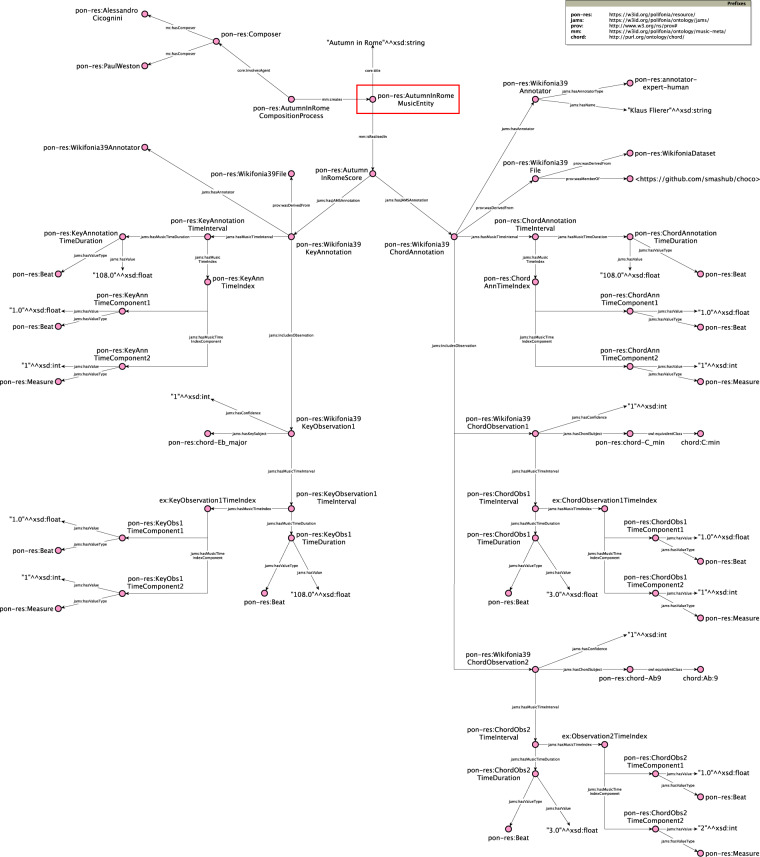
Example of data modelled using *JAMS Ontology*, extracted from a track from the *Wikifonia* dataset. The track is annotated from a score, therefore annotations and observations contain time references expressed in *beat* and *measure*.

Figure 6 shows an example of data from the *Wikifonia* subset (*wikifonia_39*) annotated using the *JAMS Ontology*. Starting from the individual highlighted by the red box (pon-res:AutumnInRomeComposition) we can trace information related to the piece entitled *“Autumn in Rome“*. The file includes two annotations (pon-res:Wikifonia39KeyAnnotation and pon-res:Wikifonia39ChordAnnotation), derived from a score (pon-res:AutumnInRomeComposition mc:hasScore pon-res:AutumnInRomeScore), hence their temporal information is expressed as a combination of beats and measures. The chord annotation (pon-res:Wikifonia39ChordAnnotation) contains two observations, the first starting at the beginning of the first measure (*measure* 1, *beat* 1), while the second starts at the beginning of the second measure (*measure* 2, *beat* 1). They both have a duration of 3 *beats*.

We remark that the music time interval of an annotation is different, though dependent on, the time interval of its observations: it must include all of them. In the example of Fig. 6, the time interval of pon-res:Wikifonia39ChordAnnotation starts from the beginning of the first measure (*measure* 1, *beat* 1) and has a duration of 108 *beats*.

A JAMS observation, according to the JAMS data model, can only have one subject (jams:hasSubject), which is a music projection (mp:MusicProjection) e.g. chord, key mode, pitch. The main musical feature currently treated in ChoCo is the chord. A chord (mp:Chord) is indeed modelled as a special type of mp:MusicProjection.

**Fig. 7:**
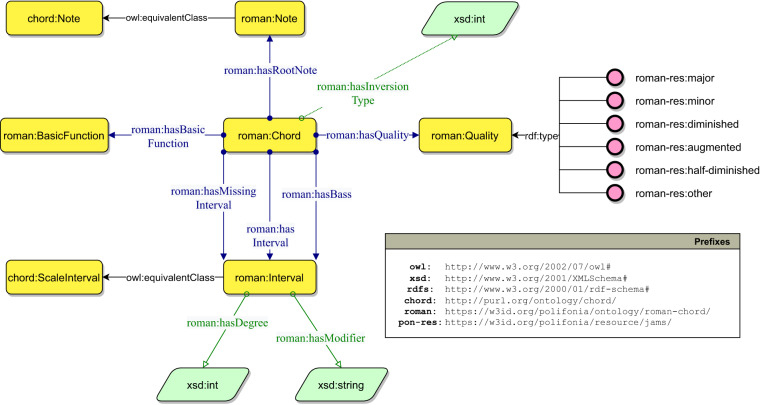
The Roman Chord Ontology describing Roman Numeral Chords and their constituting elements.

As presented in Conversion of chord notations, ChoCo focuses on two chord notations: Harte and Roman Numerals. In the JAMS Ontology, the Harte notation is addressed by reusing the Chord ontology^23^. For modelling Roman Numerals, we developed the *Roman Chord Ontology* (https://github.com/polifonia-project/roman-chord-ontology), which is part of the *Music Analysis* module in PON. Figure 7 shows the main features of the ontology, which is available at the following URI:

https://w3id.org/polifonia/ontology/roman-chord/

The core class roman:Chord defines roman numeral chords. A chord is a complex structure, therefore it is described by means of several properties. The classes roman:BasicFunction and roman:Quality describe the chord from a functional harmony perspective and the quality of the chord (e.g. major, minor, augmented), respectively. The class roman:Note describes the absolute pitch of the bass note, while the class roman:Interval is used to describe the bass, the internal intervals of the chord and any missing intervals. Each interval is described by the datatype properties roman:hasDegree, which describes the degree of the interval, and roman:hasModifier, which describes any alterations to the interval. Finally, the datatype property roman:inversionType defines the possible type of inversion of the chord.

To streamline this process and simplify its reuse, we also release service APIs allowing to generate knowledge graphs of roman numeral chords – starting from their symbol and a reference key. The API service can be queried as follows:

https://w3id.org/polifonia/resource/roman-chord/[romanChord]_[key]

**Fig. 8:**
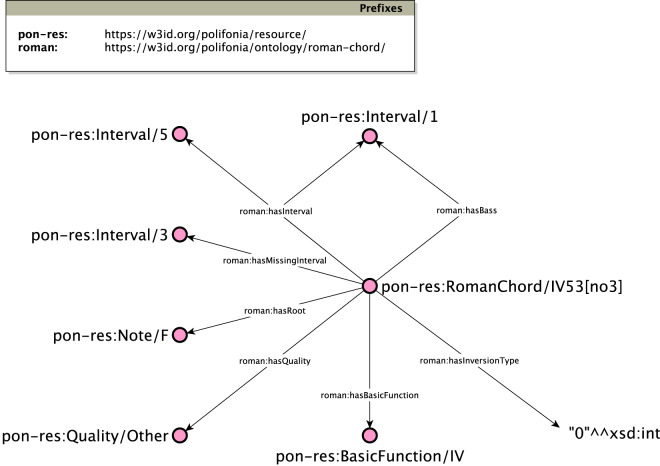
Example of a Knowledge Graph generated using the *Roman Chord Ontology* on a IV53[no3] chord.

For example, the API call https://w3id.org/polifonia/resource/roman-chord/IV53[no3]_C will produce the knowledge graph illustrated in Fig. 8.

#### Knowledge Graph construction

To build the ChoCo Knowledge Graph (ChoCo KG) we propose jams2rdf, an open-source tool to convert any JAMS file to RDF, with the following usage: jams2rdf.py <input_jams_file> [<outout_rdf_file>]. jams2rdf relies on *SPARQL Anything*^62^, a tool supporting querying with SPARQL any data from any file format. We use SPARQL Anything’s JSON module to define a SPARQL CONSTRUCT query template that generates ChoCo triples according to the JAMS Ontology (Fig. 4). This allows for a modular design, as different conceptualisations, ontologies and triplifications for JAMS can be added in separate, independent SPARQL queries. We also publish additional queries to facilitate the extract and the manipulation of specific JAMS fields from the KG.

To build the ChoCo KG, we iteratively run jams2rdf using the query template over our entire collection of curated JAMS files. This yielded ≈30 milion RDF triples. More statistics on the ChoCo KG can be found in the Melody portal of the Polifonia Project (https://projects.dharc.unibo.it/melody/choco/chord_corpus_statistics).

## Data Records

The descriptive statistics reported in this section provide an overview of ChoCo at two different levels: the metadata associated to the music tracks and scores in the dataset (the musical content being annotated), including their identifiers and links; and the actual content of the music annotations.

In ChoCo v1.0^29^ (from now on, ChoCo), the dataset contains 20,086 JAMS files: 2,283 from the audio collections, and 17,803 collected from symbolic music. In turn, these JAMS files provide 60,263 different annotations: 20,530 chord annotations in the Harte notation, and 20,029 annotations of tonality and modulations – hence spanning both local and global keys, when available. Besides the harmonic content, ChoCo also provides 554 structural annotations (structural segmentations related to music form) and 286 beat annotations (temporal onsets of beats) for the audio subsets.

### Metadata and external links

**Fig. 9:**
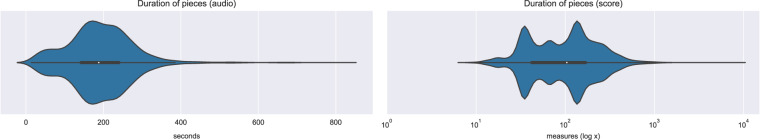
Distribution of track (*left*) and score (*right*, log-x scale) durations, measured in seconds and measures, respectively.

The average duration of the annotated music pieces is 191.29 ± 85.04 seconds for (audio) tracks; with a median of 104 measures for symbolic music, and Interquartile range ${\rm{IQR}}={\rm{Q3}}-{\rm{Q1}}=168-42=126$ (Q1, Q3 denote first and third quartiles, respectively). As illustrated in Fig. 9, this provides a heterogeneous corpus with a large extent of variability in the duration of pieces, which also confirms the diversity of musical genres in ChoCo (Table 1). For instance, a folk tune can span a few measures and still possess a musical identity with respect to the genre; in contrast, a sonata can cover hundreds of measures.

**Fig. 10:**
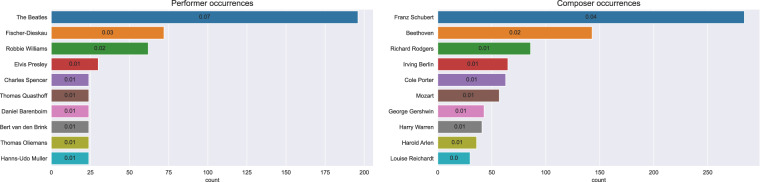
Overview of the ten most common performers (*left*) and composers (*right*) in the dataset, when explicitly distinguishable from their generic “artist” attribution in the metadata.

From the metadata extraction of the JAMification step (c.f. From chordal data to JAMS datasets), it was possible to disambiguate 2421 artists as *performers* – which represent 12.05% of the dataset, and a total of 7,304 as *composers* (36.36% of ChoCo). This implies that the remaining 51.59% of JAMS files only provide generic *artist* information (with no distinction between composers and performers), whereas another small portion of the dataset – corresponding to the JazzCorpus (0.37% of ChoCo), does not provide any metadata. An overview of the ten most common performers and composers is reported in Fig. 10, with “*The Beatles*” and “*Franz Schubert*” being the most recurring names, respectively.

The JAMS files in ChoCo also contain 771 links to other resources, representing about 3.8% of the dataset. These were extracted from the original collections, and automatically verified and corrected for validity (link/identifier working) and consistency (disambiguation of the resource pointed, e.g., musical work, recording, and release). Most links point to MusicBrainz (78%), whereas a few of them link to Wikidata (6%), IMSLP (6%), YouTube (5%), and to other datasets (5%).

In addition to these explicit links, which can already be found in the JAMS files, we also link the resources in the ChoCo KG to two other large-scale music datasets on the Web:

1. **MIDI Linked Data Cloud**^28^. The ChoCo chord annotations can be useful for harmonic analyses of existing scores and symbolic music representations, e.g. MIDI. To link MIDI URIs with ChoCo URIs, we compare the string similarity of the original MIDI filename and the JAMS file_metadata name, both typically containing the band/artist and song names, and link them through midi:midiOf if their similarity is >0.80. This yields 2,411 links. However, we do not inspect the musical content to establish this linkage, meaning that the harmonic annotation of a sonata in C minor would be linked to the same sonata in D minor, as long as their titles are highly similar. Therefore, the verification and the provision of links that are musically plausible (beyond the metadata) are currently under investigation.
2. **Listening Experience Database** (**LED**)^63^. Relating harmonic properties of pieces and their evolution to music listening experiences throughout history is also another promising direction. For those listening experiences that are explicitly associated to a musical work through dc:subject and mo:performance_of (where dc and mo prefix *Dublin Core* and *Music Ontology*, respectively), we extract links with ChoCo’s resources via text similarity of work titles (using the same criteria as before). Links can be further filtered whenever a musical work in LED also provides a reference to the artist (via mo:composer or mo:performer). Overall, this yields 1996 links.

These additional links open up new research directions, as they allow to relate harmonic content (chord changes, harmonic complexity, tension, etc.) to other musical properties that are inherently present in the music (melodic contour, expressive variations, instrumental changes, etc.), or that may have been elicited certain emotions, memories, and feelings in listeners. Here we report an example of a listening experience of *“So What”* in LED (https://data.open.ac.uk/page/led/lexp/1431335026178), which was linked to 8 chord annotations in ChoCo.

*«What do you mean by playing “without harmony“? Using a pedal tone, which Coltrane got into after a period of very dense harmonic playing. He would use one or two harmonic references throughout a song, as he did on “So What“ [from Miles Davis’s Kind of Blue, on Columbia]. It was basically D for sixteen bars, E flat for eight bars, and then back to D. Ultimately, he worked with only one harmonic reference point, and then in “Ascension“ [from Best of John Coltrane: His Greatest Years, on Impulse] there was nothing harmonically.»* (Steve Kuhn in *“The Great Jazz Pianists: Speaking of Their Lives and Music”)*

### Overview of chordal annotations

This section provides statistics on the content of chord annotations in ChoCo, their observations and temporal onsets; similar statistics can also be extracted for tonality annotations (local and global keys), but are excluded here to focus on chordal content.

**Fig. 11:**
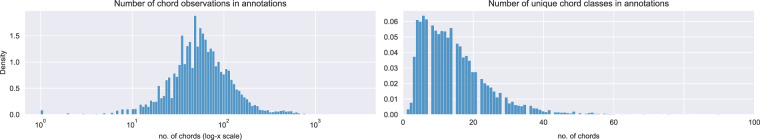
Distribution of the number of chord observations per annotation (*left*, linear scale) and their distinct chord classes (*right*, log-x scale). The latter can also be considered as the cardinality of the chord set used by each annotation.

**Fig. 12:**
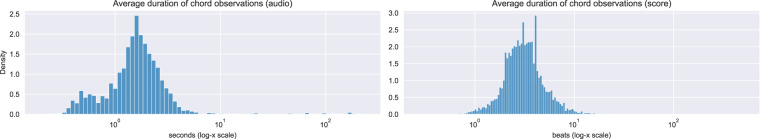
Distribution of chord durations for audio (*left*, seconds) and symbolic (*right*, beats) annotations on log-x scale.

Overall, and without any simplification/collapsing of chords, there are 1,575,409 chord occurrences/observations in ChoCo, with an average annotation having 76 chords (Fig. 11, *left*). When looking at the unique chord occurrences in the harmonic progressions (chord classes) – measuring the chordal diversity of the annotations, the dataset counts 306,407 chords, which are drawn from a set of 7,281 possible classes. An annotation, on average, uses 14.92 ± 11.10 chord classes (Fig. 11, *right*). The median duration of chord observations in audio and score JAMS is 1.6 (Q1 = 1.12, Q3 = 2.15) seconds and 3.06 (Q1 = 2.33, Q3 = 4) beats, respectively (Fig. 12). For most statistics reported in this section, we observe right skewed distributions (long tails on the right side) as negative values (e.g. negative durations) cannot occur; and we report log-x plots for convenience.

**Fig. 13:**
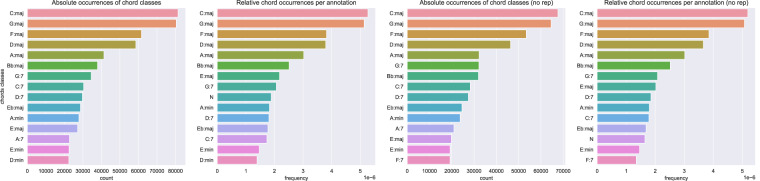
Absolute and relative occurrences of chord classes in the original annotations (*left*, *centre-left*), and after removal of consecutively repeated chords (*right*, *centre-right*). Absolute occurrences are counted and accumulated throughout the corpus, whereas relative occurrences are first aggregated per annotation, as frequencies, then averaged across the whole dataset. Note that the “N” chord class denotes the “silent chord” as per the Harte notation (obtained for all subsets).

The fifteen most common chords in ChoCo, based on their absolute and relative occurrences, are reported in Fig. 13 (*left*). Absolute counts are obtained by accumulating the chord counts for each annotation/progression across the dataset (as if all annotations refer to the same piece). Instead, relative counts are computed by first normalising the absolute counts of each annotation by the number of chord observations in the progression; then averaging the resulting chord frequencies across all annotations. Analogously, Fig. 13 (*right*) reports the same statistics after removal of consecutively repeated chords. This pre-processing step aims to mitigate consecutive repetitions (which may arise due to the different temporal granularity of chord observations, or possess a harmonic function) from inflating the chord count. Regardless of the counting method, the three most common chords in the dataset are: *C:maj*, *G:maj*, and *F:maj*.

A similar analysis is also reported for chord n-grams, which are typically used to find harmonic patterns in songs. To avoid trivial n-grams, these are computed after removal of consecutive repetitions (e.g. G:7, G:7, C:maj becoming G:7, C:maj). Table 4 ranks the first 10 n-grams based on their relative count (frequency).

**Table 4:** Summary of the most common chord n-grams (*n* = 2, 3, 4), ranked by their relative occurrence (frequency) per chord annotation.The last column reports the corresponding total number of n-gram occurrences in the dataset (no aggregation).

Order	Rank	Chord 1	Chord 2	Chord 3	Chord 4	Frequency	Occurrences
2	1	G:maj	C:maj	—	—	9.894371e-07	11560
	2	C:maj	G:maj	—	—	9.314316e-07	9968
	3	C:maj	F:maj	—	—	8.578674e-07	9837
	4	D:maj	G:maj	—	—	8.447899e-07	11229
	5	G:7	C:maj	—	—	8.270923e-07	12590
	6	G:maj	D:maj	—	—	8.236944e-07	9591
	7	F:maj	C:maj	—	—	7.588854e-07	8547
	8	D:7	G:maj	—	—	7.092709e-07	10673
	9	A:maj	D:maj	—	—	6.319998e-07	6925
	10	C:7	F:maj	—	—	6.247398e-07	10362
3	1	G:maj	C:maj	G:maj	—	4.156081e-07	4487
	2	C:maj	F:maj	C:maj	—	4.022300e-07	4167
	3	D:maj	G:maj	D:maj	—	3.518498e-07	4473
	4	C:maj	G:maj	C:maj	—	3.210295e-07	3209
	5	G:maj	D:7	G:maj	—	2.757892e-07	3411
	6	G:maj	D:maj	G:maj	—	2.755515e-07	3483
	7	C:maj	G:7	C:maj	—	2.685492e-07	3371
	8	F:maj	C:maj	F:maj	—	2.601499e-07	2660
	9	A:maj	E:maj	A:maj	—	2.201239e-07	1767
	10	A:maj	D:maj	A:maj	—	2.151695e-07	2450
4	1	G:maj	C:maj	G:maj	C:maj	1.984606e-07	1933
	2	C:maj	G:maj	C:maj	G:maj	1.897574e-07	1746
	3	C:maj	F:maj	C:maj	F:maj	1.840459e-07	1693
	4	F:maj	C:maj	F:maj	C:maj	1.759950e-07	1509
	5	D:maj	G:maj	D:maj	G:maj	1.647309e-07	2256
	6	G:maj	D:maj	G:maj	D:maj	1.609514e-07	2105
	7	D:7	G:maj	D:7	G:maj	1.587393e-07	1873
	8	A:maj	E:maj	A:maj	E:maj	1.497483e-07	998
	9	E:maj	A:maj	E:maj	A:maj	1.453102e-07	1067
	10	G:7	C:maj	G:7	C:maj	1.338413e-07	1593

To conclude, the number of chord annotations for which the identity of the annotators is known is 796 (3.9% of the dataset).

## Technical Validation

To validate the data transformation workflow presented in Methods (Fig. 1), focusing on the output of the JAMifier (generation of JAMS files from arbitrary chord collections) and the Chonverter (chord alignment and conversion) modules, we conducted two separate analyses: a groundtruth evaluation of JAMS files, and an expert validation of chord conversions.

### Validation of the JAMifier

As the goal of the JAMifier is to automatically generate a JAMS dataset given a music collection providing chord annotations and metadata in different formats, notations, and conventions, this first evaluation addresses the following question.

**Q 1** *How complete and accurate are ChoCo’s JAMS files – for metadata and harmonic annotations, after the JAMification*?

To answer this question, we carried out a series of tests to compare a sample of generated JAMS files with those that are expected from this process. This required the creation of a groundtruth dataset of JAMS files that were manually produced by two human annotators from a given template (the backbone of a JAMS file), and through manual inspection of the original collections. For example, given a sample of the Wikifonia subset, the validator was expected to fill the JAMS template by: opening the MusicXML file of each assigned piece; inserting the relevant metadata (title, composer, duration, etc.) into the appropriate fields; and finding the (Leadsheet) chord labels annotated on the score – to create a JAMS Observation out of each of them. Annotators were first instructed on the task, and a preliminary annotation trial was performed to assess their reliability. After the trial, annotators received 4 templates for each subset and produced 72 gold JAMS files in total. The corresponding JAMification output is then compared to the groundtruth to measure: (i) the coverage and the accuracy of the metadata; and (ii) the coverage and error of chord and key annotations.

For the metadata, coverage is computed as the proportion of metadata fields in the gold JAMS that can also be found in the generated JAMS, regardless of their values. For example, if *title*, *composers*, *genre*, and *duration* are the expected metadata fields for a given JAMS file, and the generated counterpart only provides records for *title* and *duration*, coverage would account for 0.5 (even if both *title* and *duration* are incorrect). To provide a complementary view, metadata accuracy of common fields is computed as the normalised Levenshtein similarity among the generated and expected values for strings; or as the relative variance from the expected value for numerical fields (e.g. duration). The accuracies are then averaged for each JAMS file.

The results of this evaluation are reported in Table 5, aggregated for each subset and separated from the identifiers that were extracted from the JAMification (e.g. MusicBrainz, Wikidata). Overall, maximum accuracy and coverage are attained for most collections, and all the possible identifiers are always extracted with no errors.

**Table 5:** Average coverage and accuracy of metadata and identifiers in the generated JAMS files, per ChoCo subset. The dash symbol denotes a subset that does not provide any identifiers.

subset	metadata		identifiers	
	coverage ↑	accuracy ↑	coverage ↑	accuracy ↑
biab-internet-corpus	0.95 ± 0.1	0.9243 ± 0.0835	—	—
billboard	1.0 ± 0.0	1.0 ± 0.0	—	—
chordify	1.0 ± 0.0	1.0 ± 0.0	1.0 ± 0.0	1.0 ± 0.0
ireal-pro	1.0 ± 0.0	1.0 ± 0.0	—	—
isophonics	1.0 ± 0.0	1.0 ± 0.0	—	—
jaah	0.8036 ± 0.0595	1.0 ± 0.0	1.0 ± 0.0	1.0 ± 0.0
jazz-corpus	1.0 ± 0.0	1.0 ± 0.0	—	—
mozart-piano-sonatas	0.875 ± 0.0	1.0 ± 0.0	1.0 ± 0.0	1.0 ± 0.0
nottingham	1.0 ± 0.0	1.0 ± 0.0	—	—
real-book	1.0 ± 0.0	1.0 ± 0.0	—	—
robbie-williams	1.0 ± 0.0	1.0 ± 0.0	—	—
rock-corpus	1.0 ± 0.0	1.0 ± 0.0	—	—
rwc-pop	1.0 ± 0.0	0.9999 ± 0.0001	—	—
schubert-winterreise	1.0 ± 0.0	1.0 ± 0.0	1.0 ± 0.0	1.0 ± 0.0
uspop2002	1.0 ± 0.0	0.9661 ± 0.062	—	—
weimar	1.0 ± 0.0	0.9878 ± 0.0243	1.0 ± 0.0	1.0 ± 0.0
when-in-rome	0.7976 ± 0.0558	0.9608 ± 0.0694	—	—
wikifonia	0.95 ± 0.1	0.95 ± 0.1	—	—

For the harmonic annotations in the JAMS files, comparison with the gold counterparts is focused on *coverage* and *error* – reported independently for times (e.g. the onset of a chord occurrence), durations (e.g. how long a chord occurrence spans), and labels (e.g. a *C:maj* chord) of the observations in each annotation. The evaluation is thus in line with the structure of an observation in JAMS’ annotations (see From chordal data to JAMS datasets and Listings 1, 2). In this case, *coverage* measures the amount of the overlap between the generated and the expected observation fields, without taking order into account (this is because an extra observation may have been inserted by the annotator, thus breaking the desired alignment for comparison). For example, if (C:maj, G:maj, D:7, F:maj) and (N, C:maj, G:maj, D:7) are the labels of a generated chord annotation and the corresponding gold, respectively, the silent chord “N” breaks the alignment of those sequences. In this case, coverage would still be 3/4, as all the other chord labels are included in generated annotation. Instead, errors are computed from a 1-to-1 comparison of fields – which are assumed to be aligned. The latter can be reported according to the unit of measure of each field: seconds and beats for *time* and *duration*, and normalised Levenshtein distance for *labels* (string values).

Table 6 reports the results of this last evaluation for both key and chord annotations, where each metric is averaged by subset (mean and standard deviation). Results show good coverage and minimum error for most subsets, thus confirming the quality of the JAMification output. An exception is the Mozart Piano Sonata collection, for which low coverage and high errors are reported for key annotations. After having manually compared the JAMS sample for this subset, we found that the observations annotated by our validators in the gold set used a different temporal granularity (e.g. merging two consecutively repeated observations and aggregating their time and duration), compared to the JAMification output. Although this affected the evaluation results, both these annotations can be deemed equivalent.

**Table 6:** Evaluation of chord and key annotations in the generated JAMS files on the test samples, reported for *times*, *durations*, and *labels* of their observations, and averaged for each subset. Coverage of observation values ranges from 0 (all the expected values are not found in the generated annotation) to 1 (all the expected values are included). Errors are given as seconds (audio) or beats (symbolic) for *times* and *durations*, respectively; and as normalised text similarities for *labels*.

subset	type	Key coverages ↑			Key errors ↓			Chord coverages ↑			Chord errors ↓		
		time	duration	label	time	duration	label	time	duration	label	time	duration	label
billboard	audio	1.0 ± 0.0	1.0 ± 0.0	1.0 ± 0.0	0.0 ± 0.0	0.0 ± 0.0	0.0 ± 0.0	1.0 ± 0.0	1.0 ± 0.0	1.0 ± 0.0	0.0 ± 0.0	0.0 ± 0.0	0.0 ± 0.0
chordify	audio	1.0 ± 0.0	1.0 ± 0.0	1.0 ± 0.0	0.0 ± 0.0	0.0 ± 0.0	0.0 ± 0.0	1.0 ± 0.0	1.0 ± 0.0	1.0 ± 0.0	0.0 ± 0.0	0.0 ± 0.0	0.0 ± 0.0
isophonics	audio	1.0 ± 0.0	1.0 ± 0.0	1.0 ± 0.0	0.0 ± 0.0	0.0 ± 0.0	0.0 ± 0.0	1.0 ± 0.0	1.0 ± 0.0	1.0 ± 0.0	0.0 ± 0.0	0.0 ± 0.0	0.0 ± 0.0
jaah	audio	1.0 ± 0.0	1.0 ± 0.0	1.0 ± 0.0	0.0 ± 0.0	0.0 ± 0.0	0.0 ± 0.0	0.95 ± 0.1	0.95 ± 0.1	1.0 ± 0.0	0.06 ± 0.13	0.06 ± 0.13	0.0 ± 0.0
robbie-williams	audio	1.0 ± 0.0	1.0 ± 0.0	1.0 ± 0.0	0.0 ± 0.0	0.0 ± 0.0	0.0 ± 0.0	1.0 ± 0.0	1.0 ± 0.0	1.0 ± 0.0	0.0 ± 0.0	0.0 ± 0.0	0.0 ± 0.0
rwc-pop	audio	—	—	—	—	—	—	1.0 ± 0.0	0.53 ± 0.45	1.0 ± 0.0	0.0 ± 0.0	0.0 ± 0.0	0.0 ± 0.0
schubert-winterreise	audio	1.0 ± 0.0	1.0 ± 0.0	1.0 ± 0.0	0.0 ± 0.0	0.0 ± 0.0	0.0 ± 0.0	1.0 ± 0.0	1.0 ± 0.0	1.0 ± 0.0	0.0 ± 0.0	0.0 ± 0.0	0.0 ± 0.0
uspop2002	audio	—	—	—	—	—	—	1.0 ± 0.0	0.3 ± 0.26	1.0 ± 0.0	0.0 ± 0.0	0.0 ± 0.0	0.0 ± 0.0
weimar	audio	1.0 ± 0.0	1.0 ± 0.0	1.0 ± 0.0	0.0 ± 0.0	0.0 ± 0.0	0.0 ± 0.0	1.0 ± 0.0	1.0 ± 0.0	1.0 ± 0.0	0.0 ± 0.0	0.0 ± 0.0	0.0 ± 0.0
biab-internet-corpus	score	1.0 ± 0.0	1.0 ± 0.0	1.0 ± 0.0	0.0 ± 0.0	0.0 ± 0.0	0.0 ± 0.0	0.95 ± 0.1	1.0 ± 0.0	1.0 ± 0.0	0.05 ± 0.1	0.0 ± 0.0	0.0 ± 0.0
ireal-pro	score	1.0 ± 0.0	1.0 ± 0.0	1.0 ± 0.0	0.0 ± 0.0	0.0 ± 0.0	0.0 ± 0.0	1.0 ± 0.0	1.0 ± 0.0	1.0 ± 0.0	0.0 ± 0.00	0.0 ± 0.0	0.0 ± 0.0
jazz-corpus	score	1.0 ± 0.0	1.0 ± 0.0	1.0 ± 0.0	0.0 ± 0.0	0.0 ± 0.0	0.0 ± 0.0	1.0 ± 0.0	1.0 ± 0.0	1.0 ± 0.0	0.0 ± 0.0	0.0 ± 0.0	0.0 ± 0.0
mozart-piano-sonatas	score	0.5 ± 0.58	0.0 ± 0.0	0.5 ± 0.58	62.55 ± 125.03	139.75 ± 83.75	0.25 ± 0.29	0.85 ± 0.3	0.88 ± 0.25	0.75 ± 0.5	0.25 ± 0.5	0.15 ± 0.19	0.15 ± 0.3
nottingham	score	1.0 ± 0.0	1.0 ± 0.0	1.0 ± 0.0	0.0 ± 0.0	0.0 ± 0.0	0.0 ± 0.0	0.75 ± 0.25	1.0 ± 0.0	1.0 ± 0.0	0.85 ± 0.6	0.0 ± 0.0	0.0 ± 0.0
real-book	score	1.0 ± 0.0	1.0 ± 0.0	1.0 ± 0.0	0.0 ± 0.0	0.0 ± 0.0	0.0 ± 0.0	1.0 ± 0.0	1.0 ± 0.0	1.0 ± 0.0	0.0 ± 0.0	0.0 ± 0.0	0.0 ± 0.0
rock-corpus	score	1.0 ± 0.0	1.0 ± 0.0	1.0 ± 0.0	0.0 ± 0.0	0.0 ± 0.0	0.0 ± 0.0	1.0 ± 0.0	1.0 ± 0.0	1.0 ± 0.0	0.0 ± 0.0	0.0 ± 0.0	0.0 ± 0.0
schubert-winterreise	score	1.0 ± 0.0	1.0 ± 0.0	1.0 ± 0.0	0.0 ± 0.0	0.0 ± 0.0	0.0 ± 0.0	1.0 ± 0.0	1.0 ± 0.0	1.0 ± 0.0	0.0 ± 0.0	0.0 ± 0.0	0.0 ± 0.0
when-in-rome	score	1.0 ± 0.0	1.0 ± 0.0	1.0 ± 0.0	0.0 ± 0.0	0.0 ± 0.0	0.0 ± 0.0	1.0 ± 0.0	1.0 ± 0.0	1.0 ± 0.0	0.0 ± 0.0	0.0 ± 0.0	0.0 ± 0.0
wikifonia	score	1.0 ± 0.0	1.0 ± 0.0	1.0 ± 0.0	0.0 ± 0.0	0.0 ± 0.0	0.0 ± 0.0	1.0 ± 0.0	0.92 ± 0.17	0.75 ± 0.35	0.0 ± 0.0	0.1 ± 0.2	0.11 ± 0.18

### Validation of the Chonverter

Following the data transformation workflow illustrated in Fig. 1, we recall that the output of the JAMification step that does not natively provide Harte chord labels undergoes an alignment/conversion process through the Chonverter. First, the *Chonverter* aligns chord labels to one of the three chord families introduced in Conversion of chord notations, namely: *Leadsheet* (Harte), *Roman*, and *Polychord*. Then, a syntactic conversion is performed on each chord class, independently, to infer the corresponding Harte label. Evaluating the output of the Chonverter can thus be formulated as follows.

**Q 2** *How accurate and musically plausible are the chord alignment and chord conversion steps?*

Conversely to the previous evaluation, addressing this question requires musical expertise and familiarity with different chord notations. Therefore, we performed a 2-step evaluation with music experts to validate the alignment and the conversion rules. Four participants with at least 5 years of musical training were recruited for this experiment. Participants were first introduced to the task, and asked to express their level of familiarity with the different chord notations, and the validation methodology. Given the nature of the validation, no personal record was recorded from participants and minimal risk clearance was granted from the Research Ethics Office of King’s College London (registration number: MRSP-21/22-32842).

**Step 1** The first step focused on validating the context-free grammars used to parse chords in the original formats and aligning them to the corresponding chord families. Participants were presented with 3 different grammars, including 250 mapping rules to validate. Whenever a rule was deemed incorrect, participants were asked to provide the expected mapping.

**Step 2** Once chords were converted, the final result of the conversion was validated. This step also allowed for the validation of other conversion types that were not validated in Step 1, such as Roman numerals and Polychords. In addition, even for annotations originally provided in Leadsheet, this step allows for the validation of added/removed notes and inversions.

The first step allowed to validate all the grammar rules used for decomposing leadsheet chords into their constituting degrees. Each grammar consists of a set of *shorthands* grouped into classes. For example, the class referring to minor chords is composed of the shorthands “m” and “min”. Each class is then mapped to the degrees that compose that type of chord: for minor chords, the degrees associated with that class are 1, *b*3, 5. This type of validation was required due to the limited musical background of the dataset’s curators. All grammar rules reported incorrect by the experts were corrected and revised. A total of 27 rules within the validated grammars were updated. The corrections were of two main types: i) *correct shorthands but incorrect degrees*: the group of shorthands assigned to degrees was correct, but the degrees into which the chord was decomposed had one or more errors; ii) *inconsistent group of shorthands*: the grouping of shorthands in classes was incorrect. In this case, the shorthand(s) not belonging to the class was moved to the correct class if it existed, otherwise a new class was created. This implies that the preliminary chord alignment of the Chonverter is potentially error free.

The second validation step consisted in distributing spreadsheets in which the original chords were shown in the first column whereas the second column showed the chords converted by the *Chonverter* module. Before starting this validation phase, all participants were provided with a thorough documentation of all types of annotation used, including Harte. Furthermore, chords annotated in the *Roman Numeral* format, which had not been validated in the previous step, were tested for the first time. Experts were asked to mark whether the conversion to the Harte format was correct or not. The evaluation results are as the percentage of corrected chords out of the total (Table 7).

**Table 7:** Evaluation of chord conversions performed by music experts on a selection of ChoCo subsets.

Subset	Validated chords	Chord type	Correct conversions	Incorrect conversions	Accuracy ↑
ireal-pro	39	leadsheet	37	2	0.949
rock-corpus	40	roman	40	0	1.000
weimar	37	leadsheet	37	0	1.000
when-in-rome	40	roman	40	0	1.000
wikifonia	40	leadsheet	39	1	0.975
*average*	196	*all*	193	3	*0.985*

## Usage Notes

The availability of a large chord dataset, providing high-quality harmonic annotations with temporal information, content metadata, and links to external resources, is of considerable interest to several research communities. In the field of MIR, chord datasets are a fundamental prerequisite for training and evaluating content-based music algorithms that can accommodate a variety of tasks – from chord recognition and cover song detection, to automatic composition systems. For musicology and computational music analysis, the scale and diversity of ChoCo^29^ would enable large scale cross-corpus studies across different musical periods, genres, and artists (e.g. uncovering potential influences), and the KG can also be leveraged to run complex queries entailing certain musicological properties of chords, rather than relying exclusively on their notation-specific label. Also the Semantic Web community would benefit from the introduction of high quality chord data that can be linked to existing Web resources. In turn, this opens up new scenarios and research opportunities for the aforementioned communities.

### First experiments in Polifonia

An example of novel application at the intersection of both SW and MIR is the Semantic Music Player that the Polifonia consortium demonstrated at the 2021 “AI and Music festival” by SONAR (https://www.starts.eu/agenda/aimusic-festival-sonar-cccb/detail/). By leveraging the semantic integration and linking of three collections in ChoCo – including pop (Isophonics), jazz (JAAH), and classical (Schubert Winterreise) music, a mobile app providing an augmented listening experience was developed on top of the resulting KG. During playback of a song, and depending on the user’s preferences and liking, the app can visualise semantic links to related music pieces, depending on controllable musical facets (e.g. harmonic and lyrics similarity) and common entities (e.g. locations, contexts of production). Whereas the harmonic similarity links are enabled by the chordal content of ChoCo, all the other connections were obtained from linking metadata information of these three subsets with MusicBrainz, SecondHandSongs (https://secondhandsongs.com/), Songfacts (https://www.songfacts.com), Genius (https://genius.com/), and Wikidata. Overall, this application provided an example of how the ChoCo KG can be leveraged for music listening and recommendation, as well as corroborating the potential of SW technologies in such domain – using ontologies to model musical content and relationships explicitly, transparently and meaningfully, as opposed to black-box AI methodologies.

Another line of research that is of particular interest to Polifonia is the creation of music similarity networks^64^, and their consequent investigation through network data analysis techniques^65^. In a music similarity network, nodes typically represent artists, composers, or music pieces (or a relevant grouping of pieces), whereas edges express content-specific relationships of similarity. In the context of ChoCo, this has already led to the design of new methods for harmonic segmentation and similarity, which in turn, are fuelling the creation and the expansion of the *Harmonic Memory*^66^. The computational analysis of the Harmonic Memory can indeed reveal interesting insights resulting from the exploration of harmonic relationships from a global perspective, building upon the local 1-to-1 relationships on which similarity is usually defined. This includes tracing potential influences between authors and across pieces^67^, identifying common harmonic patterns, discovering disruptive artists/pieces^65^, as well as providing analytical support to formulate or test musicological hypotheses. For instance, an algorithmic procedure on the Harmonic Memory may discover, or empirically support, that two authors use similar but not identical harmonic structures, even though there is no direct connection between them, but possibly through the influence of a third entity.

### Applications and tasks

Given the diversity, size, and quality of the corpus, we expect ChoCo to enable novel applications in Music Technology, other than supporting the design and the evaluation of methods addressing specific tasks in both MIR and computational music analysis. Besides the aforementioned applications in music listening and recommendation, another case study involves the advancement of systems for machine creativity. In the context of our work, these include automatic (or semi-automatic) composition, with particular focus on *arrangement generation*^68^ (generating a chord progression, possibly given a melody to accompany); and *melody generation* through harmonic conditioning^10^ (generating a melody to play along with a chord progression that is provided as a harmonic template). In ChoCo v1.0, this is enabled by the integrated *Wikifonia* and *Nottingham* collections; and in future versions, with melodic data from *Rock Corpus*, *Weimar*, and the Band-in-a-Box collections.

Not only does ChoCo support the creative capabilities of such systems – by providing a considerable amount of quality training data, but it also contributes to their automatic *evaluation*. In fact, the evaluation of music generation systems has recently attracted a growing interest in the field, due to the concerning ethical implications these tools are raising^69^. On one hand, this involves the extraction of statistical features quantifying the degree of alignment between a generated repertoire and the training material, with respect to certain musical properties^70^; on the other hand, it concerns the detection of potential sources of plagiarism in generated music within and beyond the training set^71^.

Another application domain that can benefit from the Chord Corpus is that of *music pedagogy*. For example, TheoryTab (https://www.hooktheory.com/theorytab) allows users to choose from a repertoire of popular songs and visualise their harmonic/melodic structure during playback – with chords encoded in both Leadsheet and Roman notations, and projected in such a way as to facilitate the theoretical understanding of the song. Chordify uses chord recognition systems to infer and align chord progressions from audio recordings, and provides support for practising them with guitar, piano, and ukulele. Despite their value, both the technology and the data powering these commercial tools are not openly available, thereby decreasing their overall wider use. In contrast, ChoCo provides an attractive open and linked solution, with its modular architecture enabling the semantic description of chords according to the desired level of complexity and granularity (e.g. an educational ontology for chords might provide a simpler vocabulary). This makes it more suitable for educational purposes.

In the context of MIR, the use of ChoCo can support a multitude of tasks. The nature of its contribution is twofold: (i) it provides an unprecedented amount of training data, which is often essential for the effectiveness of supervised methods; (ii) it contributes to the development of graph-based methodologies for music analysis that can leverage the semantic representation of chord progressions. For instance, a central research area in MIR is *music similarity*, which in turn encompasses a number of interrelated tasks, including *cover song detection* – useful for music cataloguing and to support court decisions in music plagiarism^72^; and content-based *music retrieval*, aiming to search scores or performances from musical repositories using either symbolic queries, singing (alias query-by-humming), or by playing a smart instrument^73^. Another example of a MIR task that would benefit from ChoCo is *music structure analysis*^74^, which is concerned with the detection and labelling of structural segments related to musical form – a task that strongly relies on the use of harmonic/melodic features^75^. Other tasks of interest include *music tagging*^76^, such as *music genre/style classification* and *composer/artist identification*. Finally, examples of tasks of musicological interest that would benefit from ChoCo include *pattern mining*, *cadence detection*, and *local key identification*.

### Online survey

Since ChoCo is a new resource for the SW, MIR and Musicology communities, we discuss here evidence for potential adoption. To gather such evidence, we performed an online survey in which we directly ask potential adopters 10 questions regarding their background, relevance, and interest in working with chord data. The online survey was distributed in the SW, International Society for MIR (ISMIR), and Digital Musicology mailing lists, gathering a total of *N* = 53 responses. The survey was conducted via Google Forms – without recording any personal data from participants or any contact information.

**Fig. 14:**
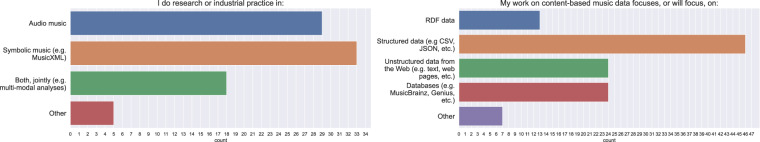
Overview of responses to Questions 2 (music domains, *left*), and 3 (data types, *right*) in the survey.

**Fig. 15:**
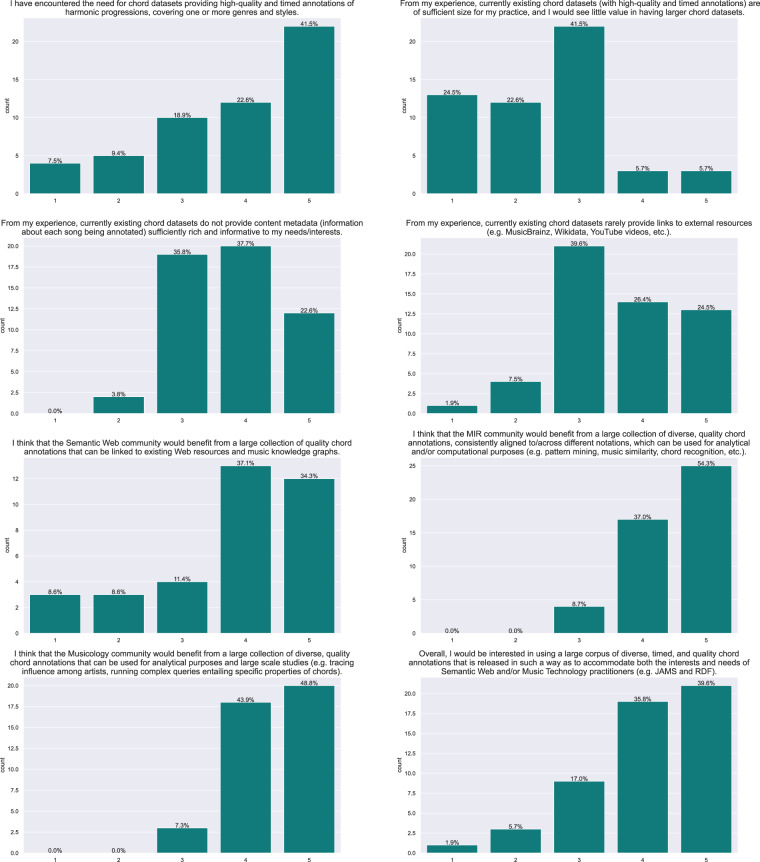
Questions and overview of responses for Questions 4–11 from the online survey.

Results are illustrated in Figs. 14, 15. Except for questions 1–3 and 12 (multiple choices), all questions ask participants to quantify the agreement with the statement made from 1 (absolutely disagree) to 5 (absolutely agree), 3 being a neutral response (neither agree nor disagree). In the first three questions we assess the background of the respondents, finding that 38 work in MIR, 27 in Musicology, 13 in Semantic Web, and 5 are also involved in other fields (AI, Music Theory, Music Interaction). Most respondents do research or industrial practice using audio (29) or symbolic music (33), or both (18), focusing primarily on structured data when conducting content-based music studies (Fig. 14). Nevertheless, music researchers also make extensive use of unstructured data and music databases, and 13 of them (24% of respondents), utilise RDF data.

From questions 4–11 we found that: 64% of respondents have encountered the need for chord datasets providing high-quality timed annotations of harmonic progressions, covering one or more genres/styles; 47% believe that currently existing chord datasets are not of sufficient size for their practise (whereas 41.5% have a neutral position); about 60.3% argue that such datasets do not provide content metadata sufficiently rich and informative to their needs (with another 35.8% being neutral); and 51% believe that links to external resources (e.g. MusicBrainz, Wikidata, etc.) are rarely provided (40% are neutral). Each research community strongly recognises the value of a dataset like ChoCo as a key resource for their field: MIR (91.3%), Semantic Web (71.4%), Musicology (92.7%), and overall, 75.4% of respondents expressed their interest in using such a dataset.

**Table 8:** Licensing per ChoCo subset. The second column details the licence declared by the data curator of the corresponding subset; it indicates “*not specified*” whenever this information was not made explicit in articles, web-pages, collection metadata, repositories, etc. The last column refers to the licence attributed to the standardisation-integration output for each subset within ChoCo – which is made compliant to the original licence, as derivative work. Please, note that all the authors of the “*not specified*” subsets were contacted to verify whether the use of a CC-BY licence was compliant to their data publishing policies.

ChoCo subset	Original licence	ChoCo licence
Isophonics	Not specified	CC BY 4.0
JAAH	CC BY-NC-SA 4.0	CC BY-NC-SA 4.0
Schubert-Winterreise	CC BY 3.0	CC BY 4.0
Billboard	CC0	CC BY 4.0
Chordify Annotator Subjectivity Dataset	CC BY-NC-SA 4.0	CC BY-NC-SA 4.0
Robbie Williams	Not specified	CC BY 4.0
Uspop-2002	Not specified	CC BY 4.0
RWC-Pop	Not specified	CC BY 4.0
Real Book	Not specified	CC BY 4.0
Weimar Jazz Database	ODbL	CC BY 4.0
Wikifonia	public domain	CC BY 4.0
iReal Pro	public domain	CC BY 4.0
Band-in-a-box	Not specified	CC BY 4.0
When in Rome	CC BY-SA 3.0	CC BY 4.0
Rock Corpus	CC BY 4.0	CC BY 4.0
Mozart Piano Sonata	CC BY-NC-SA 4.0	CC BY-NC-SA 4.0
Jazz Corpus	Not specified	CC BY 4.0
Nottingham	Not specified	CC BY 4.0

## Data Availability

The ChoCo dataset and Knowledge Graph, together with the ontological ecosystem and code, are publicly available from several repositories (c.f. Table 2). As detailed in Methods, ChoCo is currently released in 2 modalities: • As a JAMS dataset, where audio and score annotations are distinguished by the type attribute in their Sandbox; and temporal/metrical information is expressed in seconds (for audio) and measure:beat (for scores) ; • As a Knowledge Graph, based on our JAMS ontology to model music annotations, and on the Chord and Roman ontologies to semantically describe chords; Table 2 also provides links to a live SPARQL endpoint. We have implemented a number of actions to ensure that these outputs are in compliance with the FAIR Guiding Principles for scientific data management and stewardship^21^. A GitHub repository hosts data, code, and instructions (https://github.com/smashub/choco), to fully reproduce the corpus creation from the original collections. To improve reproducibility, the repository also provides a Docker image for the project (platform agnostic). To improve data consistency, both the latest versions of ChoCo (JAMS file and RDF triples) are available on Zenodo, in synchronisation with GitHub releases. Via GitHub and Zenodo, the ChoCo project has a unique and persistent identifier and is registered in a searchable source. Additionally, via our integration framework, ChoCo contains fine-grained provenance descriptions that allow to keep track of the original source of each harmonic annotation – both in terms of annotators (the person who contributed the harmonic analysis) and data curator (the maintainer of the original collection). Finally, to comply with the original collections, all data and code in ChoCo is released under the *Creative Commons Attribution 4.0* licence (CC-BY 4.0), with the exception of the JAAH, CASD, and Mozart Piano Sonata subsets – which follow the *Creative Commons Attribution-NonCommercial-ShareAlike 4.0* international licence (CC-BY-NC-SA 4.0). This required an in-depth analysis of the licensing policies of the integrated collections (see Table 8). Indeed, for 7 collections, we could not find any specific licensing information from related scientific articles, technical reports, online resources, repositories, dataset metadata, and so forth. For these cases, the authors of these collections were contacted and confirmed whether the use of the CC-BY 4.0 licence – on our derivative integration work – was compatible with their original releasing strategies.
